# Blending Ethnomedicine with Modern Technology—From Conventional to Tailored Products: Modulating Biopharmaceutical Properties of *Berberis* Extract by Solid Lipid Nanoparticles for Wound Healing

**DOI:** 10.3390/jfb14080418

**Published:** 2023-08-09

**Authors:** Neetika Sharma, Karan Vasisht, Jasmine Kaur, Simarjot Kaur Sandhu, Kaustav Dey, Bakr Ahmed Hameed, Rakesh Bajaj, Indu Pal Kaur, Maninder Karan

**Affiliations:** 1 University Institute of Pharmaceutical Sciences, Panjab University, Chandigarh 160014, India; 2 Nanz Med Science Pharma (P) Ltd., Paonta Sahib 173025, India

**Keywords:** herbal, natural extract, drug delivery, solubility, stability, excision wound, scar formation, phytopharmaceutical, nanophytopharmaceutical, controlled release

## Abstract

Drug-delivery systems employing phytopharmaceuticals based on the leads in traditional knowledge offers not only an alternative but quicker and more economic strategy for drug development. Nanophytopharmaceuticals promise remarkable opportunities with the ability to overcome challenges associated with herbal medicines, such as low solubility and bioavailability, poor target specificity, and shelf life. *Berberis* extracts documented as Ropana (wound healer) in *Sushruta Samhita* are a popular traditional remedy that is amiss in the modern system of medicine as it exhibits very poor biopharmaceutical properties. Poor solubility and bioavailability necessitate the administration of high doses to achieve the desired therapeutic effects. Exploiting the diversified type of compounds with pleiotropic properties present in *Berberis*, the biopharmaceutical properties were engineered using an optimized freeze-dried extract and developed solid lipid nanoparticles (SLNs) as an effective drug-delivery system. An industrially viable and environment-friendly hot high-pressure homogenization technique led to a stable formulation with an average particle size of 178.4 nm, as well as a 7-fold increase in loading and a significant entrapment of 91 ± 1.25%. The pharmacodynamic studies of developed nanosystems in excision-wound models showed faster and complete healing of wounds with no scars.

## 1. Introduction

Nanotechnology, a multidisciplinary scientific undertaking, is making progressive developments on various fronts with plant actives and extracts as among the potential candidates [1]. The concept of natural synergism in plant drugs due to the presence of pleiotropic, multi-targeted molecules is gaining much significance and attention from the scientific world [2,3]. Nano-carrier systems enhance the therapeutic potential of the plant drug by overcoming the major associated issues such as low solubility and bioavailability, high-dose toxicity, the degradation of active constituents, and poor stability [4,5]. Moreover, the nanoparticulate and nano-encapsulated systems provide a controlled release, as well as a targeted delivery, of the active constituent(s) present in the plant extract [4]. The considerable attention that has been focused on the nanocarrier systems for plant active and extracts has led to the successful development of nanosystems of many prized phytochemicals and herbal drugs such as paclitaxel, silymarin, curcumin, *Ginkgo biloba*, and ginseng [6,7], among others. Herein, in contrast to the traditional processes, the industry uses technology that closely resembles the conventional processes of herbal preparations but adopts newer technologies [8,9] and produces the desired effect in mitigating various disorders [2].

*Berberis* extract is stated as *Ropana*, which is a wound healer in *Sushruta Samhita*, as the extract possesses an inherent synergistic combination of constituents with wound-healing properties [10]. It is a multicomponent anti-infective and, more particularly, an antibacterial agent credited with many more biologically useful activities [10,11]. *Berberis* extract is a potential candidate for development as a nanophytopharmaceutical because, despite wonderful uses mentioned in traditional systems of medicine and proven pharmacological effects, it is not available clinically in the modern system of medicine owing to poor solubility and bioavailability [12,13]. Further, it results in another key issue of high doses to exhibit the desired efficacy [14,15].

Many reports are available with improved physicochemical parameters of the major *Berberis* alkaloid berberine, with enhanced efficacy against CNS disorders, wounds, and cancer [16,17], but very few attempts have been made to develop a novel delivery system of *Berberis* extract with desired biopharmaceutical properties. A couple of nanoformulations have been patented for polyherbal products containing *Berberis* [18,19]. Taek Kwan et al. successfully introduced water soluble extract *B. koreana* (Korean barberry) into monoolein cubic phases [20]. Ansar et al. synthesized silver nanoparticles with the root bark of *B. lycium* [21]. However, the nanosystems of *Berberis* developed so far suffer from potential drawbacks of low drug loading, poor absorption, or the use of organic solvents [19]. The multiple uses and bioactivities of *Berberis* have always intrigued pharmacologists and clinicians, but until now, it has not evaded the attention of formulation scientists for improving its biopharmaceutical properties to the extent of making it clinically available. Therefore, the present study (patent filed) [22] blends the ethnobotanical uses of a single *Berberis* extract with modern nanocarrier systems and designs it in a way that results in a novel, cost-effective, patient-friendly, safe, simple, and industrially amenable formulation that exhibits efficient and fast wound healing.

## 2. Materials and Methods

### 2.1. Plant Material

The stem samples of *Berberis lycium* were collected from the surroundings of St. Bede’s College, (31.0938° N, 77.1868° E), Shimla, Himachal Pradesh, India. The identity of the plant sample was authenticated by National Institute of Science Communication and Information Resources (NISCAIR) vide reference no. 3283-84-2. The sample specimens were deposited at Museum–cum–Herbarium of University Institute of Pharmaceutical Sciences, Panjab University, Chandigarh with voucher number 1484. The plant material was shade dried, cut into slices, crushed in an electric grinder to obtain the moderately fine powder, and stored in freezer at −20 °C in polyethylene bags for further experiments.

### 2.2. Chemicals and Reagents

The solvents used were from E. Merck (India) Ltd. (Mumbai, India). LR grade solvents were used for extraction, and chromatographic grade was used for chromatographic analysis. Type II deionized water produced by Milipore system was used for preparing the solutions and dilutions. Compritol^®^ 888 ATO was a gift sample from Gattefosse, France, and Phospholipon 90 G (soya lecithin) was gifted by Lipoid, Germany. All other chemicals, reagents, and reference standards were from Sigma-Aldrich Chemicals Pvt. Ltd., Bengaluru, India.

### 2.3. Preparation of Berberis Extract

The *Berberis* extract was prepared by extracting *B. lycium* stem following optimized extraction conditions using Design-Expert Software.

The powdered plant material was extracted by reflux method using optimized conditions of extraction (communicated elsewhere) with 80% aqueous ethanol (1:25) under acidic pH for 150 min. The residue obtained from the extract was dried to remove last traces of solvent by two techniques: vacuum oven and freeze dryer. The two differently dried extracts were examined and characterized using optical microscopy, Fourier transform–infrared spectroscopy (FT–IR), hot-stage microscopy (HSM), differential scanning calorimetry (DSC), thermogravimetric analysis (TGA), and powder X-ray diffraction (PXRD).

### 2.4. Spectrophotometric Analysis of Berberine

Standard plots of berberine were prepared in three different solvents: (a) Methanol; (b) Mixture of choloroform:methanol (1:1); and (c) Mixture of ethanol:phosphate buffer of pH 6.8 (40:60) for spectrophotometric analysis in different matrices. Stock solutions of berberine were prepared in each of the three solvents by dissolving an accurately weighed quantity of berberine in the respective solvent in a volumetric flask. The stock solutions were serially diluted to obtain six working concentrations. The absorption of each of these working solutions was recorded at 345 nm (λ_max_ of berberine) against a blank of respective solvent in UV-visible spectrophotometer. The observed absorbance of each working solution was plotted against its concentration, and the best fit standard curve of straight line forced through zero was constructed. The obtained equation of regression line was used to calculate the attenuation coefficient, $E_{1\mathrm{cm}}^{1\%}$ of drug.

### 2.5. Preparation of Berberis Extract-Loaded Nanoformulation

The freeze-dried *Berberis* extract with higher solubility in desired solvents was processed for developing nanoformulation as solid lipid nanoparticles (SLNs) using hot high-pressure homogenization technique.

#### 2.5.1. Preparation of SLNs by High-Pressure Homogenization

*Berberis* extract (1 g) was dissolved in PEG 400 (8 g). The lipid, Compritol^®^ 888 ATO (4 g), was melted separately. Phospholipon 90 G (0.4 g), tween 80 (8 g), and water (78.6 mL) were combined and heated to the lipid-melting temperature of 82 °C. The dissolved *Berberis* extract was next added to the lipid phase. The hot-lipid phase containing *Berberis* extract was dropped all at once into the aqueous phase using a high-speed stirrer (WiseTis HD 15D, Wertheim, Germany) at a speed of 8000 rpm for 15 min. The coarse emulsion thus formed was passed thrice through a high-pressure homogenization (HPH) using the homogenizer (Emulsiflex C3 Avestin) at a pressure of 1000 bars, and three cycles were run. The mixture was cooled to room temperature and later shifted to a refrigerator. It was observed for 4 weeks to maintain the physical form. In case any deposit or crystals were observed, the formulation was discarded. The blank SLNs were prepared following the same procedure but without adding the freeze-dried *Berberis* extract.

#### 2.5.2. Preparation of *Berberis* Extract SLNs Gel

Compritol^®^ 888 ATO 1.5 g was dispersed in 10 mL of water and kept overnight for swelling. Triethanolamine was added drop-wise to this mixture with continuous stirring to affect the gelling of carbopol. Stirring was continued until a translucent gel was formed. The dispersion of freeze-dried *Berberis* extract-loaded SLNs (88.5 mL equivalent to 0.885 *Berberis* extract/g of gel) was added to the prepared gel and mixed slowly to obtain a homogeneous mixture. Blank SLN gel was prepared similarly by adding lyophilised blank SLN dispersion (88.5 mL) into the prepared gel.

#### 2.5.3. Characterization of SLNs

##### Optical Microscopy

SLNs of *Berberis* extract were observed under optical microscope at 40× magnification after suitable dilution in distilled water to record morphology of SLNs.

##### Field Emission Scanning Electron Microscopy (FESEM)

It was performed in an electron microscope of Hitachi High-Technologies, Europe, model SU8010. The dispersion was placed on Nucleopore Track–Etch membrane and silicon wafer was attached to the dried membrane followed by sputter coating with platinum. The sample was observed at 140 °C and a voltage of 15 kV and images were captured.

##### Particle Size and Polydispersity Index (PDI)

Mean diameter and polydispersity index were measured in a 10× diluted dispersion using laser diffraction with particle size analyzer of Beckman Coulter, Brea, CA, USA model, DelsaTM NanoC.

##### Total Drug Content (TDC)

A known quantity of SLN dispersion was disrupted by adding a mixture of chloroform:methanol (1:1) to it in a tube and vortexing the mixture. The vortexed mixture was filtered to obtain a clear solution. The absorption of the clear filtrate was recorded at 345 nm for berberine (obtained as the major active constituent of *Berberis* extract) against a blank of chloroform:methanol (1:1).

##### Entrapment Efficiency (EE)

Entrapment efficiency of the SLNs of freeze-dried *Berberis* extract was determined using dialysis membrane method. The amount of berberine in the receptor medium was estimated spectrophotometrically and obtained as the estimate of unentrapped *Berberis* extract. Meanwhile, the SLN remaining inside the bag following dialysis was disrupted as explained above (in the TDC section) to obtain a direct estimate of the entrapped *Berberis* extract obtained as berberine.

##### In Vitro Release Studies

In vitro release studies were performed using Franz diffusion assembly. Dialysis membrane was soaked in double distilled water for 12 h prior to use [23]. A volume of 30 mL of release medium (mixture of ethanol:phosphate buffer of pH 6.8) was added to each cell, and the temperature was maintained at 37 °C with continuous stirring throughout the experiment. The dialysis membrane was loaded with SLN dispersion and was placed over receptor cell containing release medium. Aliquots of release medium from the cell were withdrawn at pre-determined time intervals until more than 90% drug release was accomplished. The volume of release medium in the cell was kept constant by replenishing the medium after each sample collection. The collected samples were suitably diluted and analyzed in UV-visible spectrophotometer.

##### Zeta Potential

It was measured for a 10× diluted SLN dispersions using high concentration cell in Beckman Zetasizer at 25 °C and electric field strength of 23.2 V/cm.

##### Fourier Transform Infrared Spectroscopy (FT–IR)

The FT–IR spectra were recorded on Agilent Technologies FT–IR, model 630 Cary using Micro Lab Software. Samples of freeze-dried *Berberis* extract, freeze-dried *Berberis* extract-loaded SLNs, Compritol^®^ 888 ATO, phospholipon 90 G, and physical mixture of Compritol^®^ 888 ATO with PEG 400 melted and solidified were analyzed over a range of wave number 400–4000 cm^−1^.

##### Differential Scanning Calorimetry (DSC)

The DSC thermographs were obtained by taking 2–5 mg of the sample in a crimp aluminum pan. The DSC model Q20 of TA Instruments, New Castle Delware, DE, USA was used in the analysis. DSC thermographs of freeze-dried *Berberis* extract, freeze-dried *Berberis* extract-loaded SLNs, and Compritol^®^ 888 ATO were recorded over a temperature range, which varied from 30 °C to 350 °C at a heating rate of 10 °C/min.

##### Hot-Stage Microscopy (HSM)

The dried extract samples were mounted in hot-stage microscope fitted with computer-controlled programmable hot stage, polarizing filters, and digital camera for recording thermal events. The samples were heated from 25 °C to 230 °C at a defined heating rate. Melting points and physical changes in the extract samples were visually examined at 50× magnification, and the images of thermal events were captured in camera.

##### Thermogravimetric Analysis (TGA)

TGA was performed on a Mettler Toledo TGA/SDTA 851θ instrument. Approximately 5 mg of dried extract sample were heated from 25 °C to 300 °C in an open alumina pan at a heating rate of 10 °C/min under nitrogen purge at a flow rate of 50 cc/min. The thermal changes in the samples were recorded, and a plot of percentage weight loss versus temperature was obtained to analyze the behaviour of the samples.

##### Powder X-ray Diffraction (PXRD)

PXRD was performed using XPERT–PRO diffractometer (PANalytical, Almelo, The Netherlands) with a CuKα radiation (1.54060 A°). *Berberis* extract-loaded SLNs, Compritol^®^ 888 ATO, phospholipon 90 G, and physical mixture of Compritol^®^ 888 ATO with PEG 400 melted and solidified were studied for PXRD.

##### Rheological Studies

Rheological profile of the freeze-dried *Berberis* extract SLN gel was performed using a rotational type rheometer (Rheolab QC, Anton Paar, Graz, Austria) attached with a water jacket (C-LTD80/QC) for maintaining constant temperature.

##### pH

The pH of *Berberis* extract-loaded SLN gel was measured using L1–120 pH meter (Elico, Mumbai, India).

##### Texture Analysis

The texture analysis for firmness and stickiness of freeze-dried *Berberis* extract-loaded SLN gel was conducted using TTC spreadability rig fitted on Texture AnalyzerTM (M/s Stable Micro Systems Ltd., Godalming, UK).

### 2.6. In Vivo Wound Healing Activity

Excision wound-healing model in mice was used to assess the therapeutic efficacy of the formulation. Male Lacca mice weighing 25 ± 3 g were procured from the Central Animal House facility of Panjab University vide IAEC approval no. PU/45/99/CPCSEA/IAEC/2018/181. The mice were anaesthetized with single intraperitoneal administration of ketamine HCl (87 mg/kg in combination with xylazine). The dorsal skin was shaved and full-thickness round, skin wound of nearly 5 mm in diameter was created using a disposable skin-punch equipment for wounds [24,25].

#### 2.6.1. Treatment

A total of seven groups (each *n* = 6) consisting of two control groups, naïve and positive control (wound without treatment), and five test groups were included in the study. The test groups comprised *Berberis* extract SLN gel, *Berberis* extract, blank SLN gel, berberine gel, and marketed drug (Soframycin). The respective samples were applied on the skin wounds of mice twice a day for 14 days. The observations were made for visual changes starting from initiation of treatment until the final day of observations. After 14 days, mice were sacrificed by cervical dislocation of anesthetized animals. The skin flaps were dissected (1 × 1 cm portions) for biochemical estimation and histopathology.

#### 2.6.2. Biochemical Estimations

A 10% (*w*/*v*) homogenate of skin sample was prepared, and the supernatant was used for the estimation of protein using biuret method [26], lipid peroxidation according to method of Wills [27], superoxide dismutase (SOD) levels according to the method of Kono [28], catalase by method of Luck [29], and reduced glutathione levels according to method described by Ellman et al. [30].

#### 2.6.3. Histopathological Analysis

At the end of experiment, a 1 cm × 1 cm tissue was collected from the center of the healed area of the randomly chosen animal of each group. The tissue was fixed by immersion in 10% formalin. The transverse sections of 3–5-µm tissue were prepared from each group and stained with haematoxylin–eosin to reveal the cellular structure. The tissues were observed under microscope to study histopathological changes.

### 2.7. Statistical Analysis

Graph Pad Prism was used for statistical analysis of data obtained from biochemical studies.

## 3. Results

### 3.1. Preparation of Berberis Extract

The powdered plant material was extracted by reflux method using optimized conditions of extraction (communicated elsewhere) with 1:25 ratio of 80% aqueous alcohol under acidic conditions for about 150 min. The extract was filtered, and the marc was washed with fresh hot-extraction solvent. The solvent from the combined extract was recovered in a rotary vacuum evaporator to obtain about 10% of the residue. The method of drying is known to influence the physical and chemical characteristics of the final product. Thus, the residue obtained from the extract was dried to remove last traces of the solvent by two methods: a vacuum oven, and the freeze-drying method. The dried extracts were sealed and stored in deep freezer at −20 °C until further use. The dried extracts were compared for their solubility, which is an important and relevant character for formulation development and bioavailability. The characterization of the vacuum oven-dried and freeze-dried extracts using methods such as FT–IR, DSC, HSM, P–XRD, and TGA revealed (Figures S1−S7) the differences in the nature of the two differently dried extracts. DSC thermograms together with TGA of vacuum-dried sample showed broad and sharp peaks, indicating melting process. This event was accompanied by weight loss of 70%. However, only broad peaks were observed in freeze-dried sample indicating lack of crystallinity. In vacuum oven-dried sample, decomposition accompanied the melting process of crystalline components. This was also well-supported by hot-stage microscopy of two samples. The semi-crystalline nature of vacuum oven-dried sample was further indicated by P–XRD, as few sharp peaks depicted the semi-crystalline nature of the constituents. These peaks disappeared in freeze-dried sample clearly highlighting that freeze-dried sample was more amorphous than vacuum oven-dried sample. The solubility of the freeze-dried extract (obtained under particular conditions of freeze-drying), in hydrophilic solvents/surfactants increased significantly by more than 20 folds as compared to vacuum oven-dried extract. Therefore, enhancement of solubility of freeze-dried extract was achieved by changing its physical form from semi-crystalline to amorphous.

### 3.2. Development of Nanoformulation of Berberis Extract

The marketed formulations of *Berberis* extract are mostly polyherbal in the form of either cream, gel, tincture, or dried herb. It not only has poor penetration through the biological membrane, but its oral dose at 1000 mg/kg/day [31] is also a very high dose, and oral bioavailability is reportedly very low [32]. Therefore, there is a need to circumvent its poor solubility and bioavailability by presenting it in a solubilized form with increased bioavailability. The development of lipid nanoformulation was considered one such powerful way to achieve this goal. In addition, the lipid nanoformulation can allow a stable formulation with sustained and target specific delivery with decreased dose, improved efficacy, and low toxicity [33].

#### Preparation of Freeze-Dried *Berberis* Extract-Loaded SLNs

Freeze-dried *Berberis* extract-loaded SLNs were prepared using hot high-pressure homogenization technique. Compritol^®^ 888 ATO was chosen as the lipid component as it expressed more solubilization in comparison with other screened lipids. Polyethylene glycol (PEG 400) was used as a co-solvent and surfactant as it effectively dissolved the freeze-dried *Berberis* extract. Coating with PEG, a polymer of hydrophilic nature, expressed greater results as PEG has high hydrophilicity, chain flexibility, and electrical neutrality, and it lacks functional groups, avoiding undesired interactions with the biological components, which is very important in topical formulations. The composition of the formulation was optimized by selecting only those formulae where nanoparticulates remained homogenously dispersed on storage. Preparations showing any mass or crystals were discarded. The selected stable dispersion (exhibiting high assay and encapsulation efficiency) was then incorporated into a gel base used as a secondary vehicle for the ease of application.

### 3.3. Characterization of SLNs

**Figure 1:**
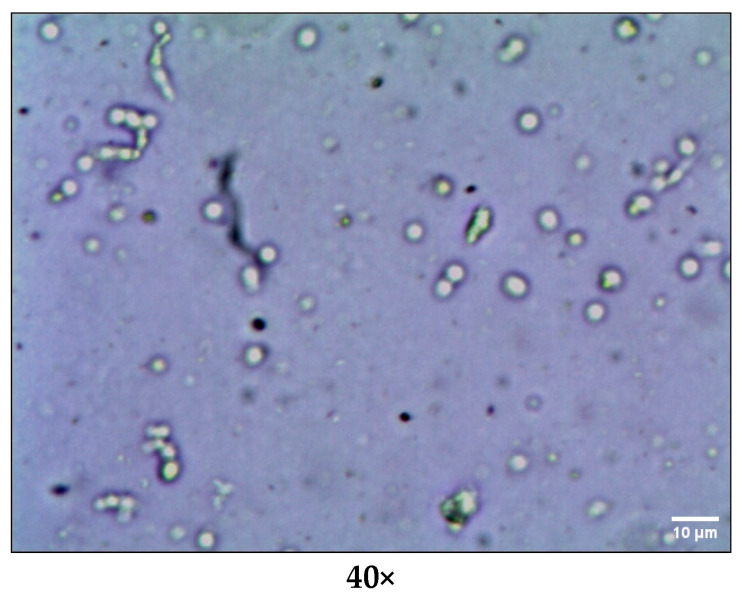
Optical microscopy of freeze-dried *Berberis* extract-loaded SLNs.

**Figure 2:**
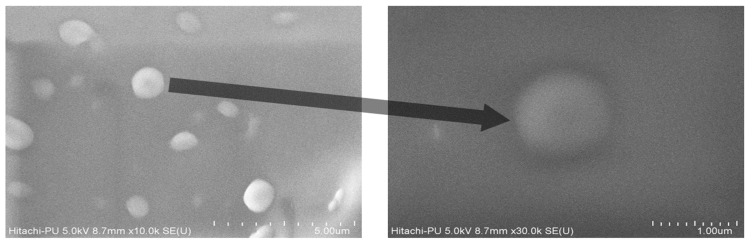
FESEM of freeze-dried *Berberis* extract SLNs. Arrow represents magnified image of that one nanoparticle.

**Figure 3:**
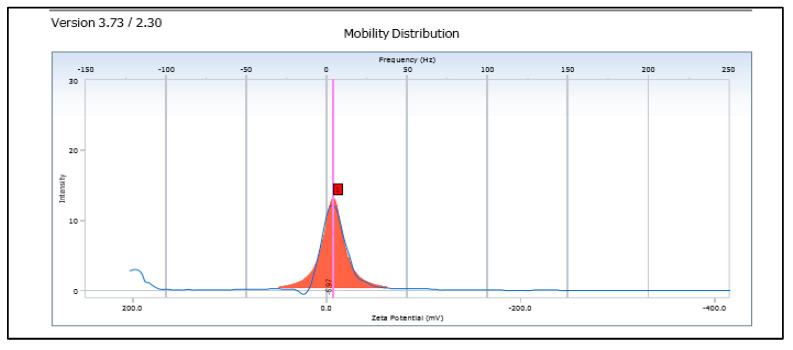
Zeta potential of freeze-dried *Berberis* extract SLNs.

**Figure 4:**
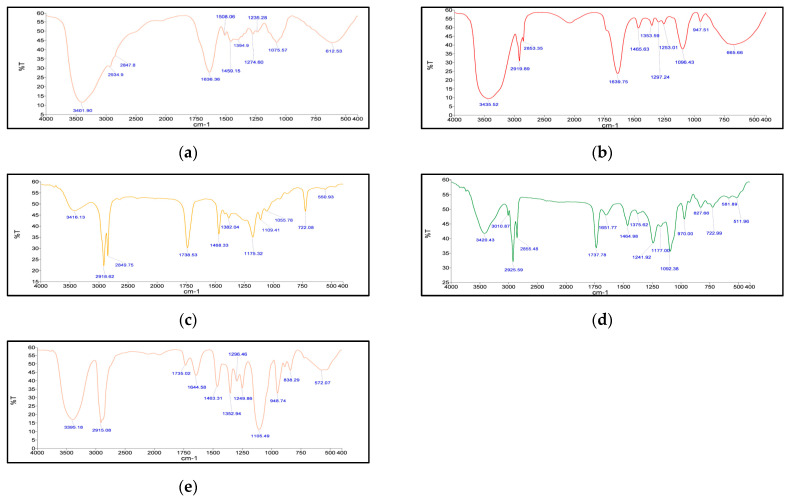
FT-IR spectra of (**a**) Freeze-dried *Berberis* extract; (**b**) Freeze-dried *Berberis* extract SLNs; (**c**) Compritol^®^ 888 ATO; (**d**) Phospholipon 90 G; and (**e**) Physical mixture of Compritol^®^ 888 ATO and PEG 400 (melted and solidified).

**Figure 5:**
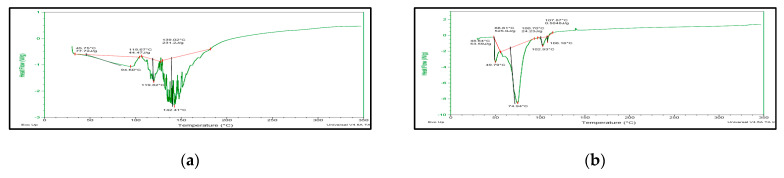

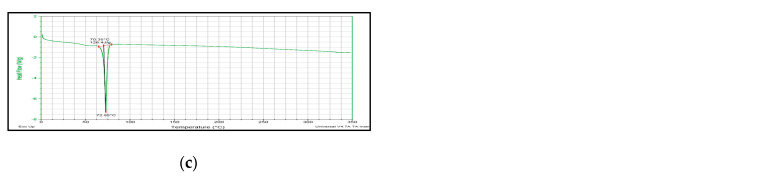
DSC thermograms of (**a**) Freeze-dried *Berberis* extract; (**b**) Freeze-dried *Berberis* extract SLNs; and (**c**) Compritol^®^ 888 ATO.

**Figure 6:**
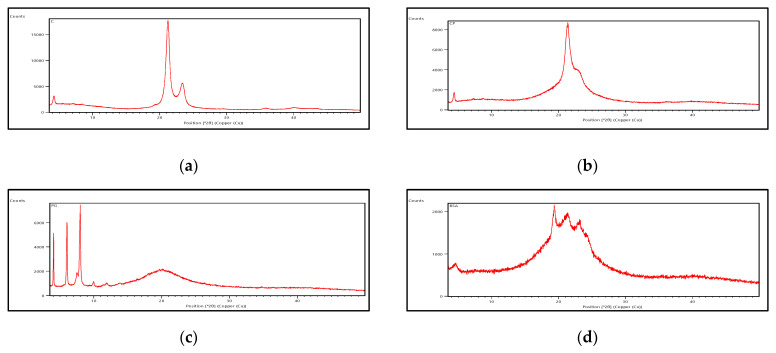
PXRD of (**a**) Compritol^®^ 888 ATO; (**b**) Compritol^®^ 888 ATO melted with PEG 400; (**c**) Phospholipon 90 G; and (**d**) Freeze-dried *Berberis* extract SLNs.

**Figure 7:**
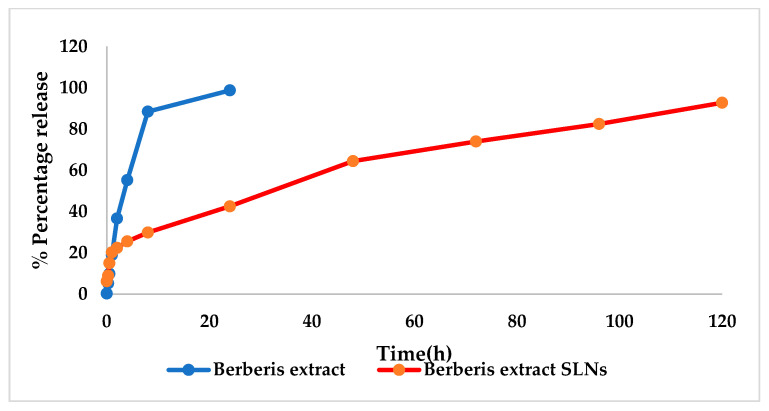
In vitro drug release from *Berberis* extract SLNs and *Berberis* extract.

The freeze-dried *Berberis* extract-loaded SLNs were subjected to all essential characterization studies. The general optical inspection indicated that the SLNs are small and round in shape, with no aggregation or irregularities (Figure 1). FESEM depicted particles that were spherical in shape and in the nanoscale range (Figure 2). The developed SLNs showed an average particle size of 178.4 nm with an average polydispersity index (PDI) of 0.289 (Figure 3). Even after 8 months of storage in a refrigerator at 3–4 °C, there was no increase in the particle size, confirming a stable nanoformulation. A drug assay (total drug content) of 86.23 ± 1.20% indicated an efficient method of production with low drug and extract loss. Entrapment efficiency (EE) of SLNs was significantly high at 90.56 ± 1.25%. Higher EE indicated the suitability of the composition and method of preparation of SLNs of the extract [34]. The observed zeta potential of developed SLNs was 6.97 mV; earlier near neutral zeta potential indicates stability [35]. The FT–IR spectrum of un-encapsulated freeze-dried *Berberis* extracts showed characteristic absorption bands at 1274.6 and 1076 cm^−1^ due to C–O–C asymmetrical and symmetrical stretching, as well as C–O stretching vibrations, respectively. Compritol^®^ 888 ATO showed characteristic peaks at 2919 cm^−1^ and 2850 cm^−1^ (alkane stretch), as well as 1738 cm^−1^ (ethers stretch). All characteristic absorption bands of freeze-dried *Berberis* extracts and Compritol^®^ 888 ATO were observed in freeze-dried *Berberis* extract SLNs with a reduction in the sharpness of some peaks (Figure 4). The FT–IR spectra of melted and solidified Compritol^®^ 888 ATO with PEG 400 showed that there was no significant interaction between the two, even during heating as both retained their characteristic peaks. This confirmed that both these components retain their individual properties when incorporated in nanoparticles, thereby indicating compatibility of the ingredients. DSC thermograms (Figure 5) of freeze-dried *Berberis* extract, freeze-dried *Berberis* extract SLNs, and Compritol^®^ 888 ATO were obtained to investigate the melting behaviour of crystalline materials. DSC thermogram of freeze-dried *Berberis* extracts showed melting point endotherms at 119.8 °C. Compritol^®^ 888 ATO showed a sharp endotherm at 72.8 °C, while freeze-dried *Berberis* extract-loaded SLNs showed broad peaks at 74.9 °C with a disappearance of the peaks observed in un-encapsulated freeze-dried *Berberis* extracts. The disappearance of the peaks indicated their efficient entrapment in the lipid matrix. The PXRD pattern of Compritol^®^ 888 ATO showed sharp peaks at 2θ scatter angles 21.19 and 23.29, indicating its crystalline state (Figure 6). Compritol^®^ 888 ATO melted with PEG 400 (21.4 and 22.8) showed pattern of peaks similar to that exhibited by Compritol^®^ 888 ATO alone. This further confirmed the FT–IR results showing no interaction between PEG 400 and Compritol^®^ 888 ATO. Freeze-dried *Berberis* extract SLNs showed broad and diffused peaks with low intensities, indicating their amorphous form in the formulation. The in vitro release profile (Figure 7), expressed in terms of the major alkaloid berberine, indicates a burst release of about 20% berberine for the first hour, 25% in 4 h, and 30% in 8 h, followed by a controlled release up to 5 days at which time 100% berberine was released. Burst release is considered to be an optimal mechanism of delivery in several instances. It is of high significance as it has been shown that many drugs, such as those used at the beginning of wound treatment, an initial burst provides immediate relief followed by prolonged release to promote gradual healing [36]. Further, part of the burst release is attributable to 10% unentrapped free extract (EE in SLNs is 90%) that was not removed from the SLN dispersion.

### 3.4. Characterization of Gel Formulation Incorporating SLN Dispersion

**Figure 8:**
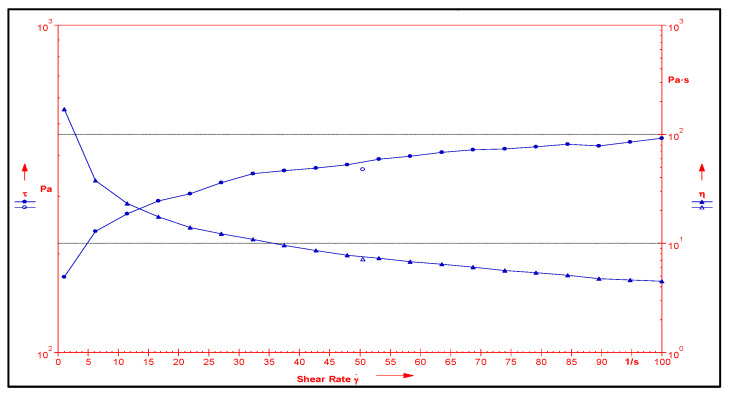
Rheological profile of *Berberis* extract-loaded SLN gel depicting relationship between shear rate, viscosity, and shear stress.

**Figure 9:**
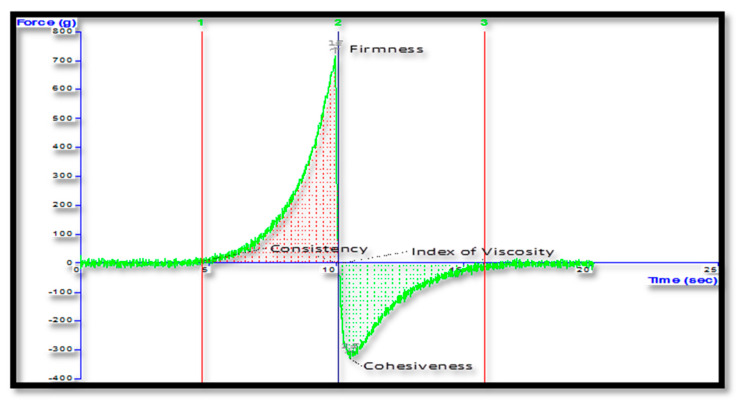
Texture analysis plot of freeze-dried *Berberis* extract SLN gel.

**Figure 10:**
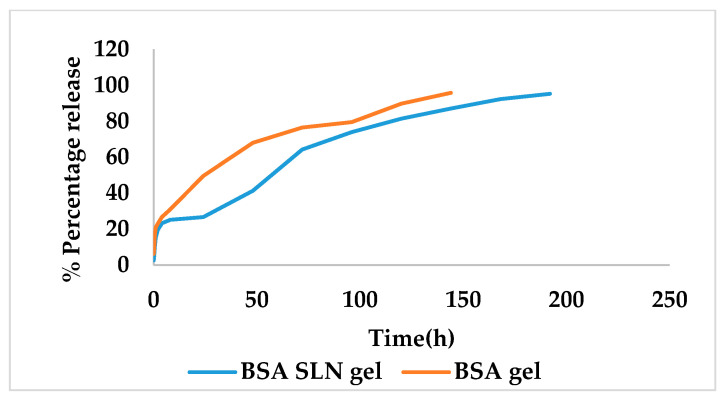
In vitro drug release from the gels of freeze-dried *Berberis* extract SLNs (BSA SLNs) and *Berberis* extract (BSA).

The rheological profile was obtained by measuring the shear rate at different shear-stress values (Figure 8). Shear thinning is a desirable property of topical gels as it facilitates the ease of application [37]. The viscosity of the formulation decreased with an increase in shear stress, suggesting a shear-thinning system. The decrease in viscosity with an increasing shear rate also suggested a non-flocculated system with a small particle size, indicating the stability of the system [38]. The texture analysis of the formulation showed a firmness of 718 g, a consistency (work of shear) of 1035.54 g.s, a cohesiveness (extrusion force) of −330.39 g, and an index of viscosity (work of adhesion) as −724.09 g.s (Figure 9). It revealed that the developed gel formulation of freeze-dried *Berberis* extract SLNs exhibited fairly good strength, ease of spreading, and extrusion from the container. It also possessed adequate cohesiveness, which is usually essential to hold the formulation at the site of action [39]. For the observed pH of freeze-dried *Berberis* extract SLNs, the gel was 5.8 ± 0.5, which was very near to the skin pH, a feature ideally needed for healthy skin. It retained pH towards the acidic side, and a controlled release favoured the pH to remain on the acidic side, which is a significant factor in controlling microbial infections, especially in chronic wounds [40]. The in vitro drug release from freeze-dried *Berberis* extract SLN gel was studied at 37 ± 0.5 °C and is shown in Figure 10. The berberine release of 95% (major active marker of *Berberis* extract) from freeze-dried *Berberis* extract SLNs was extended over 192 h. For comparison, the drug release from freeze-dried *Berberis* extract gel (using non-SLNs form) was also determined, and it showed a 96% drug release (calculated as berberine) in 144 h. This showed that the incorporation of the extract in the SLNs delayed their release, which is beneficial for extended pharmacological activity. The initial burst release provides desired loading followed by a sustained release for prolonged pharmacological effect, a phenomenon particularly desired in wound treatment for immediate relief, followed by gradual but faster healing.

### 3.5. Acute Dermal Irritation Studies

**Figure 11:**
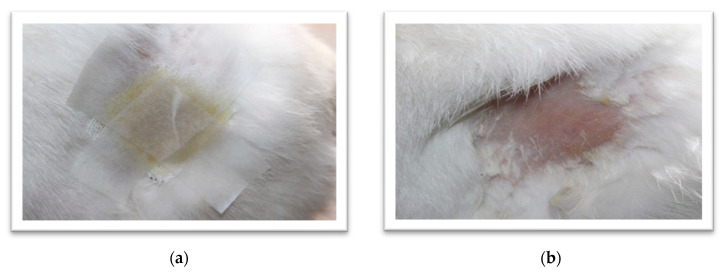
Rabbit-skin images (**a**) with formulation applied and (**b**) animal skin after application (72 h) of freeze-dried *Berberis* extract-loaded SLNs.

The developed nanoformulation was evaluated for any topical toxicity by studying acute dermal irritation to the skin as per OECD Guidelines 404. It did not cause any irritation to the dermal tissues, and Figure 11 demonstrates the test animal before and after the application of formulation.

### 3.6. Excision-Wound Model

**Figure 12:**
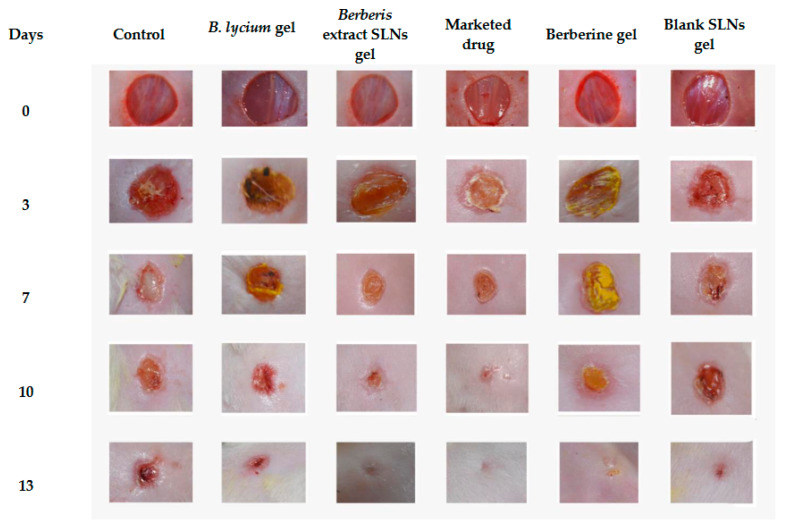
Effect of different treatment groups on the excision wound on different days.

The wound-healing potential of freeze-dried *Berberis* extract-loaded SLN gel and *Berberis* extract gel was assessed in comparison to the marketed product (Soframycin: 1% *w/w*) using the excision-wound model in mice. Representative images of the reduction in the excision-wound size with the passage of time (0, 3, 7, 14, and 18 days) are depicted in Figure 12. The wound healing with the freeze-dried *Berberis* extract-loaded SLNs gel was faster and better than the non-SLN gel of the same extract. A significant wound contraction showing a reduction in inflammation with the initiation of re-epithelialization and remodeling of the skin was clearly visible on Day 7 in these two groups in comparison to all other treatments. The developed SLN formulation gel showed 100% wound healing, as did the marketed product of framycetin (Soframycin^®^).

### 3.7. Biochemical Investigations

a. Protein estimation

**Figure 13:**
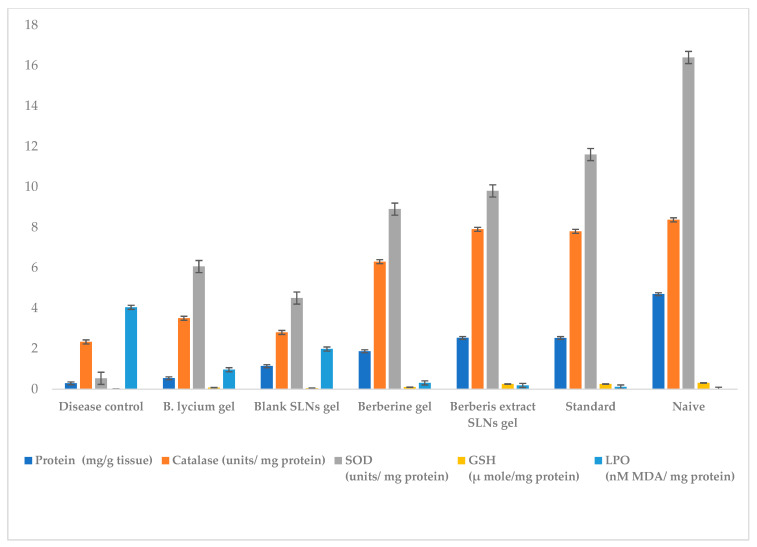
Effect of different treatment and control groups on different biochemical estimations. Data are represented as mean ± SD. Statistical significance was determined by ANOVA followed by Tukey HSD (*p* < 0.05). (For protein content, GSH, Catalase, and LPO: *Berberis* extract SLN gel was similar to standard drugs, and all other groups showed significant differences. For LPO, berberine gel was also similar to *Berberis* extract SLN gel and standard drugs.).

Protein content decreased considerably in the disease control group as compared to the naïve group with a distinct enhancement in test groups (Figure 13). The treatment groups showed significantly different activity from the disease control group. The protein content with freeze-dried *Berberis* extract-loaded SLN treatment improved by a high of 42%, as compared to the *Berberis* extract gel treatment, and was very close to the Soframycin treatment, further showing 20% improved healing over berberine gel. The central role of the protein is directly related to collagen formation in wound healing and is indicated by its increased content in healed tissue [41].

b. Measurement of free radical and antioxidant level

An ample generation of free radicals (lipid peroxidation), coupled with indigenous antioxidant imbalance (catalase, superoxide dismutase, and reduced glutathione) induces oxidative stress, tissue damage, and delayed wound healing. Therefore, a decrease in free radicals and an increase in antioxidant content is an indicator of chronic wound healing [42]. The superoxide dismutase (SOD), glutathione (GSH), and catalase levels were significantly decreased in the disease control group in comparison to the naïve group. All treatment groups in the diabetic wound-excision model showed significantly different activity from the disease control group as depicted in Figure 13. The SOD increased by 65% in the case of freeze-dried *Berberis* extract-loaded SLN gel, and 77% for the marketed standard (*p* < 0.05) compared to the disease control group. Freeze-dried *Berberis* extract-loaded SLN gel showed significantly different SOD levels versus the *Berberis* extract gel by 27%.

GSH increased by 73% and 74% in the case of freeze-dried *Berberis* extract-loaded SLN gel and the marketed standard (*p* < 0.05), while the *Berberis* extract gel and blank SLN gel showed an increase in GSH by 22% and 13% respectively, as compared to the disease control group. Freeze-dried *Berberis* extract-loaded SLNs gel versus the *Berberis* extract gel showed significantly different activity. The freeze-dried *Berberis* extract-loaded SLN gel showed a remarkable increase of 52% over the *Berberis* extract gel.

The catalase increased by 76% in the case of freeze-dried *Berberis* extract-loaded SLN gel and 75% in the case of the marketed standard (*p* < 0.05), as compared to the disease control group. However, the *Berberis* extract gel showed an increase of the catalase by just 16%, while the blank SLN gel showed an increase of only 7% as compared to disease control group. Freeze-dried *Berberis* extract-loaded SLNs gel showed significantly different catalase levels with a notable increase by 60% versus *Berberis* extract gel.

The lipid peroxidation (LPO) levels significantly increased in the disease control group in comparison to the naïve group. All treatment groups in the diabetic wound-excision model showed significantly different activity from the disease control group, as depicted in Figure 13. A remarkable decrease in the LPO level by 83% in the case of freeze-dried *Berberis* extract-loaded SLN gel, and 84% in the case of the marketed standard (*p* < 0.05) compared to the disease control group indicated faster and complete healing. In the case of *Berberis* extract gel, LPO decreased by 66%, while blank SLN gel showed a 44% decrease compared to the disease control group showing a delayed recovery over nanoformulation.

### 3.8. Histopathological Studies

**Figure 14:**
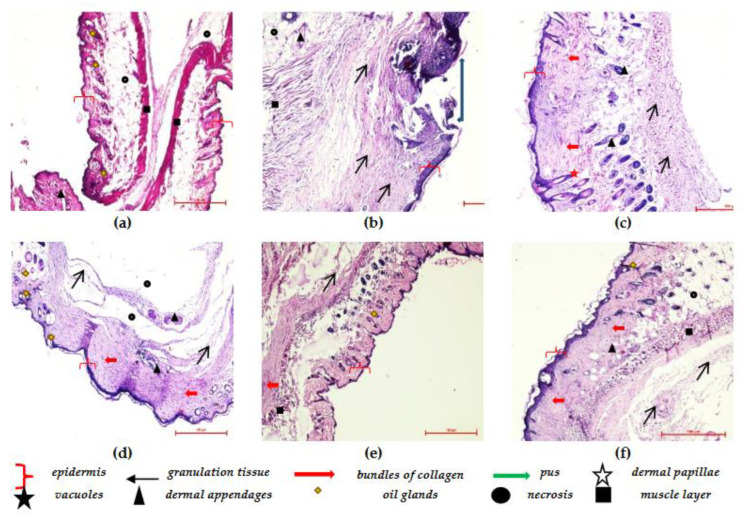
Photomicrographs of skin sections of treatment groups in excision-wound model; (**a**) Group 1: Naïve; (**b**) Group 2: Disease control; (**c**) Group 3: Blank gel; (**d**) Group 4: *B. lycium* gel; (**e**) Group 5: Berberine gel; (**f**) Group 6: Freeze-dried *Berberis* extract–SLN gel.

Histopathological examination provides comprehensive and direct evidence of tissue characteristics and any changes associated with it. It leads to a supportive wealth of information from analysis and conclusion perspectives. Hematoxylin and eosin-stained sections of skin were studied to understand the cutaneous architecture in treated groups (Figure 14). The skin sections of the naive group showed a normal constitution of the skin from the epidermis to the subcutaneous tissue. The epidermis was well-organized in multiple cell layers, along with numerous dermal papillae. Bundles of collagen fibers and other dermal appendages such as hair follicles and skeletal muscle tissue were also observed. In the case of the control group, partial healing was observed, along with the presence of fibrous tissue scars with deep ulcers. The inflammatory cells were also observed with signs of early fibrous scarring. The skin section of the *Berberis* extract showed a presence of mast cells, fibroblasts, and some healing in all the skin layers, along with inflammation. The skin was also thinned and lost hair follicles. The gaps in the epidermis of blank SLN gel groups showed superficial ulcers. The skin section showed full thickness and scarring inflammation; no edema, dense spindle cell fibroblasts, and skin appendages were observed in the wound area. Furthermore, an active inflammation with signs of early healing was observed, along with increased lymphocytes and fibroblasts. In berberine gel-treated wounds, complete healing was observed, along with oedema. Improved and fast healing was observed in the freeze-dried *Berberis* extract–SLN gel group as less nucleated fibrous tissues and more mature collagen were observed. In the case of the soframycin^®^ group, the epidermis and dermis were fairly normal with little inflammation and mild edema in the deeper dermis.

## 4. Discussion

The traditional use of medicinal plants was the first line of treatment long before the prehistoric period, and wounds are among the earliest-known ailments. *Berberis* extracts documented as *Ropana* (wound healer) is a popular traditional remedy documented in *Sushruta Samhita.* However, due to poor solubility, bioavailability, and high dose, it could not enter the modern system of medicine in the clinical setup. The current study offers blending traditional knowledge with modern technology by developing a tailored product with optimum biopharmaceutical properties and an industrially amenable SLN formulation. The results and data of the present study clearly demonstrate that the SLN formulation encapsulating freeze-dried *Berberis* extracts possess a definite pro-healing action in wound treatment. The animals treated with the nanoparticulate system exhibited a fast and improved healing of wounds over the native extract-treated group (7 days versus 10 days). The complete absence of any scar (total recovery) was seen in the SLN-treated group on Day 13, while free-*Berberis* extract-treated animals still showed incomplete healing. A distinct reason for this difference in efficacy is poor solubility. Hence, the bioavailability of *Berberis* extract as optimum therapeutic levels could not reach the target site. The nanostructure of plant extracts enhances their bioavailability, controls their release in the form of sustained delivery at the wound site, and enhances their permeability to the underlying skin layers, which are all necessary for the healing process.

The development of the novel nanosystem started with the use of an extract based on optimization technology, followed by changing the form of the extract from vacuum-dried to freeze-dried, leading to an enhanced (7-fold) increase in solubility. This was reported by us for the first time (communicated elsewhere). This formed the basis of efficient entrapment and high drug loading with a high total drug content in the developed SLNs. The excellent loading achieved in this study has not been reported so far in any of the previously published reports and patents. Further, *Berberis*, with an inherent synergistic combination of components, displayed a plethora of pleiotropic activities, working via complementary mechanisms for the complete and fast healing of wounds, leaving no scar behind. Alkaloids are the major pharmacologically active compounds of *Berberis*. Their excellent antimicrobial and anti-inflammatory action, combined with pH regulation properties, mean they possess the capacity to scavenge oxygen and free radicals. The anti-inflammatory effect is one of the first steps desired in the healing process to manage pain and inflammation. Alkaloids are known to alter the wound surface pH, stimulating wound healing via angiogenesis, collagen formation, and immunological response, specifically, macrophage recruitment and, above all, the prevention of infection in the wounds [43]. The skin-protective mechanisms via the quenching of free radicals further aid in maintaining the wound pH and reducing inflammation through the inhibition of cellular and humoral immune responses, which support the initial set-up of the healing process [44].

The phenolic components present in *Berberis* exhibit anti-lipid peroxidation potential besides neutralizing free radicals. These antioxidant properties of phenolics further improve oxygen levels and help to keep the pH lower for faster healing and preventing infections. Moreover, the polyphenolics are reported to increase epithelialization, angiogenesis, collagen deposition, and granulation, which could be key in the healing of wounds [44]. It is significant to note that the SLN formulation of the freeze-dried *Berberis* extract also exhibited a prevention of dermal scarring from wounds, which has great aesthetic and cosmetic importance. Further, histopathological analysis, a powerful acknowledged tool, obtains direct evidence of the alterations in wound-tissue etiology. The histological examination in the current study correlated the biochemical and observational evidence supporting improved and faster healing in the SLN-treated group versus other groups, especially the plain extract-gel group. The chemistry of *Berberis* and relevant biochemical analysis strongly supports the results presented in this work. A holistic look at the complete data strongly favors the clinical usefulness and a promising nanophytopharmaceutical formulation of the future.

## 5. Conclusions

Wound formation is a complex phenomenon and requires an equally composite treatment to initiate and promote all stages of healing without the fear of infections and drug resistance. Traditional therapies offer wonderful solutions but are largely not available clinically due to their poor solubility, bioavailability, high doses, and frequent stability issues. The judicious integration and development of traditional remedies into modern medicines for wound care require a stewardship that can only be gained through accurate and accessible tools and technology to offer novel, cost-effective, patient-friendly, safe, simple, and industrially amenable formulations exhibiting efficient and fast wound healing. This theory was successfully applied to modulate the poor biopharmaceutical properties of the well-known *Berberis* extract through the nanoparticulate system. The current study offers developing SLNs, a tailored product from the preparation of a suitable form of extract to developing and evaluating novel drug-delivery systems with desired properties. The use of single herb *Berberis* was preferred over polyherbal mixtures making use of assorted activities displayed by a variety of phytoconstituents contained in this herb for the healing of wounds. A blend of activities routing through different mechanisms and leading to the effective therapy of a fast and complete healing of wounds, besides preventing infection, offer a huge benefit in sharpening the adequacy of monotherapy when administered as a controlled-release novel drug-delivery system.

## 6. Patents

PCT Application number: PCT/IN2022/050106 and Indian Patent Application No. 202111005303.

## Data Availability

All relevant data is included in Supplementary File.

## Supplementary Materials

The following supporting information can be downloaded at: https://www.mdpi.com/article/10.3390/jfb14080418/s1, Figure S1: Optical microscopy of *Berberis* extract; Figure S2: FT-IR spectra of vacuum dried and freeze-dried *Berberis* extract; Figure S3: DSC curves of vacuum dried and freeze-dried *Berberis* extract; Figure S4: X-ray diffraction spectra vacuum dried (RD) and freeze-dried *Berberis* extract (FD); Figure S5: HSM images of vacuum oven-dried *Berberis* extract at different temperatures; Figure S6: HSM images of freeze-dried *Berberis* extract at different temperatures; Figure S7: TGA of vacuum dried and freeze-dried *Berberis* extract.
