# Supplementary material for: Blending Ethnomedicine with Modern Technology—From Conventional to Tailored Products: Modulating Biopharmaceutical Properties of Berberis Extract by Solid Lipid Nanoparticles for Wound Healing
Source: J Funct Biomater. 2023 Aug 9;14(8):418. doi: 10.3390/jfb14080418 (PMC10455672; doi:10.3390/jfb14080418)
Supplement: Supplementary file 1 [file jfb-14-00418-s001.zip › jfb-2465929-supplementary.pdf]

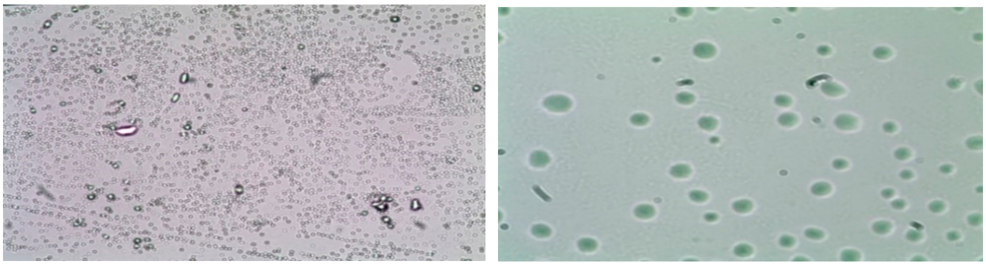

10x 100x

Freeze-dried *Berberis* extract

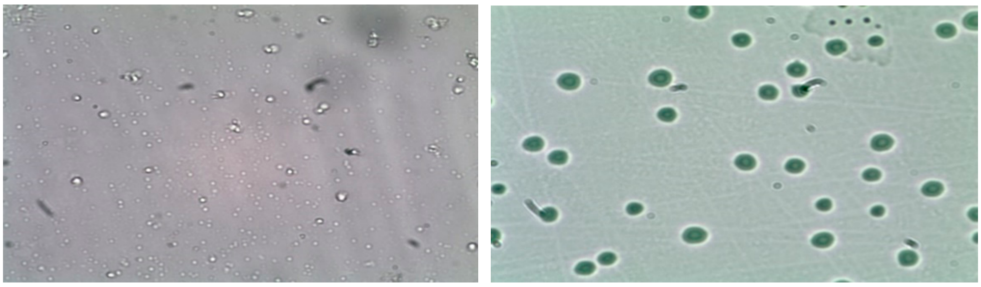

10x 100x

Vacuum oven-dried *Berberis* extract

Figure S1: Optical microscopy of *Berberis* extract

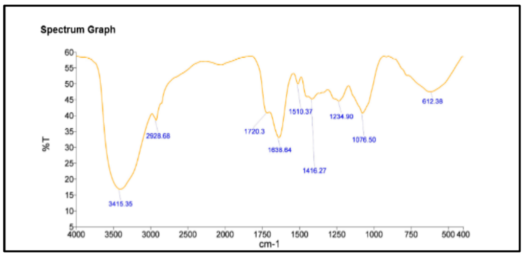


i. Vacuum dried extract

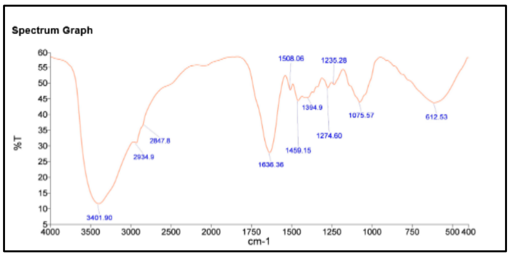


ii. Freeze-dried extract

Figure S2: FT-IR spectra of vacuum dried and freeze-dried *Berberis* extract

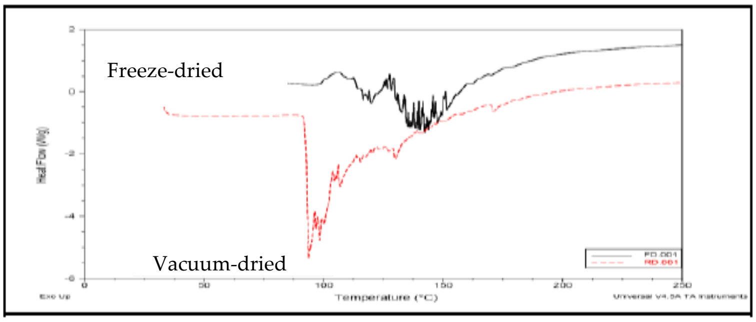


Figure S3: DSC curves of vacuum dried and freeze-dried *Berberis* extract

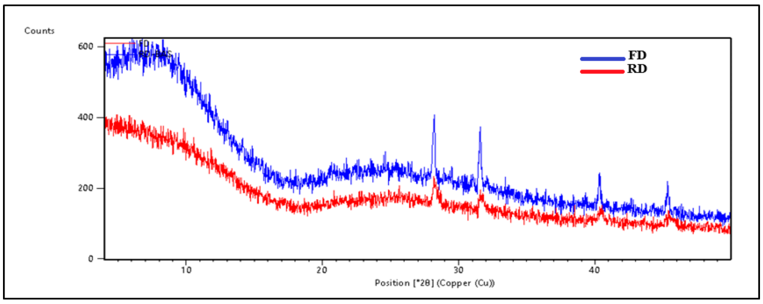


**Figure S4: X-ray diffraction spectra vacuum dried (RD) and freeze-dried *Berberis* extract (FD)**

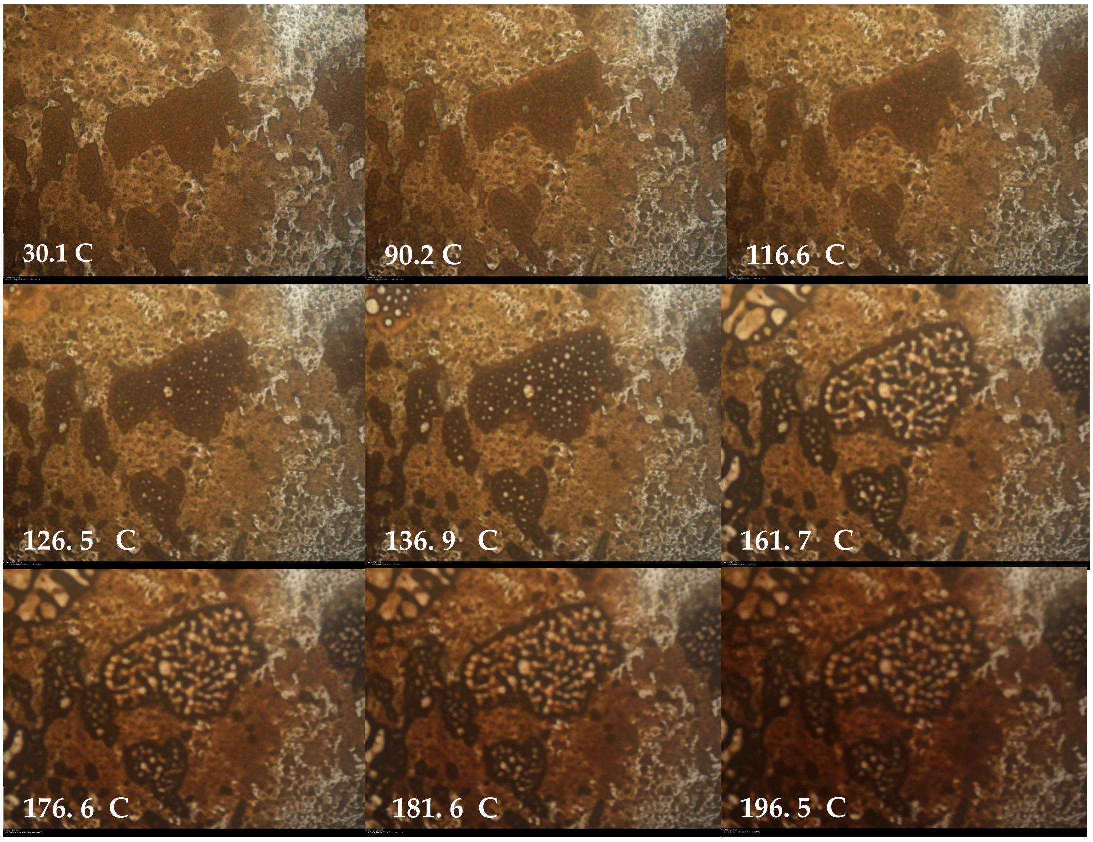


**Figure S5: HSM images of vacuum oven-dried *Berberis* extract at different temperatures**

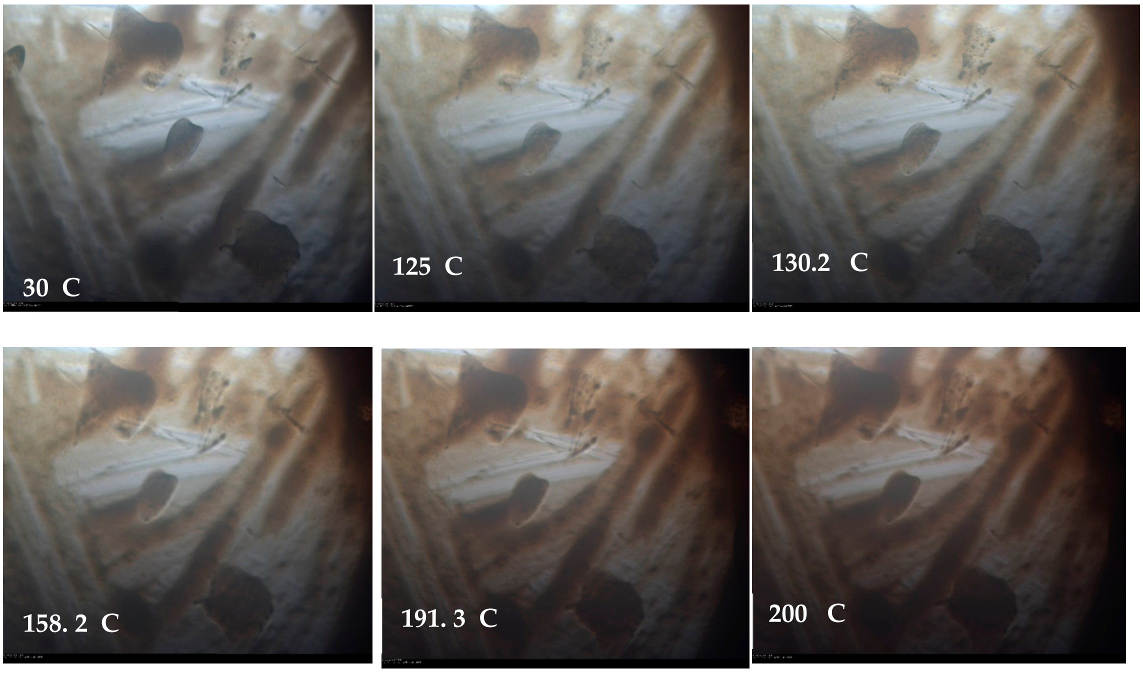


**Figure S6: HSM images of freeze-dried *Berberis* extract at different temperatures**

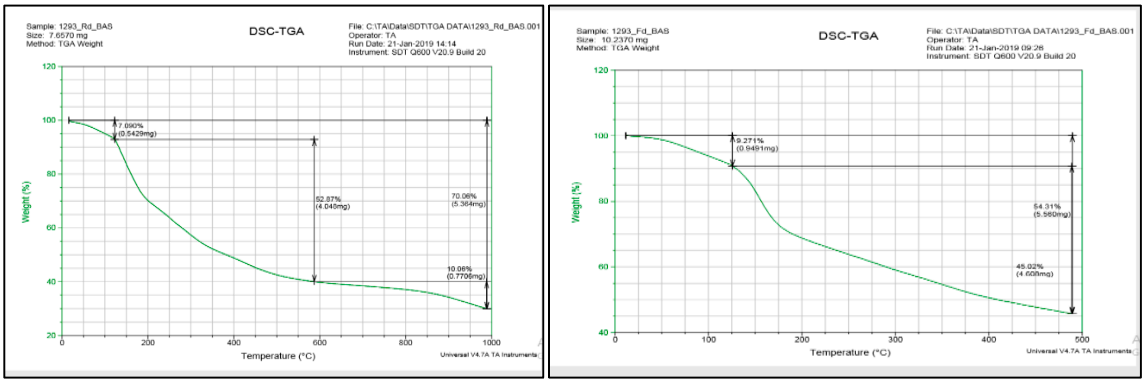


**i. Vacuum dried extract** **ii. Freeze-dried extract**

**Figure S7: TGA of vacuum dried and freeze-dried *Berberis* extract**
